# Brain-derived neurotrophic factor increase during treatment in severe mental illness inpatients

**DOI:** 10.1038/tp.2016.227

**Published:** 2016-12-13

**Authors:** G L Nuernberg, B Aguiar, G Bristot, M P Fleck, N S Rocha

**Affiliations:** 1 Hospital de Clínicas de Porto Alegre, Postgraduate Program in Medical Sciences: Psychiatry, Universidade Federal do Rio Grande do Sul, Porto Alegre, Brazil; 2 Laboratory of Molecular Psychiatry, Hospital de Clínicas de Porto Alegre, Porto Alegre, Brazil

## Abstract

Meta-analytical evidence suggests that brain-derived neurotrophic factor (BDNF) is altered in various psychiatric disorders. However, meta-analyses may be hampered by the heterogeneity of BDNF assays, lack of BDNF standard values and heterogeneity among the populations included in the studies. To address these issues, our study aimed to test, in a ‘true-to-life' setting, the hypothesis that the serum BDNF level is nonspecifically reduced in acute severe mental illness (SMI) patients and increases during inpatient treatment. Consecutive samples of 236 inpatients with SMI and 100 healthy controls were recruited. SMI includes schizophrenia and severe mood disorders, and is characterized in the sample by the presence of at least 2 years of psychiatric treatment and disability. Generalized estimating equations were used to analyze BDNF serum levels at admission and upon discharge controlled by confounding factors. BDNF levels increased significantly between admission and discharge in SMI patients. BDNF levels showed significant reductions compared with controls both at admission and upon discharge. In addition, BDNF levels showed no difference among SMI patient diagnostic subgroups (unipolar depression, bipolar depression, schizophrenia and manic episode). The increase but non-restoration of BDNF levels, even with the general acute improvement of clinical scores, may reflect the progression of the disorder characteristically seen in these patients. BDNF levels could be considered as a marker for the presence of a nonspecific psychiatric disorder and possibly a transdiagnostic and nonspecific marker of disease activity.

## Introduction

Severe mental illness (SMI) encompasses a wide range of psychiatric disorders, including schizophrenia and severe mood disorders.^1^ A suggested definition of SMI from the National Institute of Mental Health^2^ is based on the following two criteria: (1) duration, characterized as involving ‘prolonged illness' and ‘long-term treatment', operationalized as a ⩾2-year history of mental illness or treatment and (2) disability, which includes dangerous or disturbing social behavior, moderate impairment in work and non-work activities, and mild impairment in basic needs.^3, 4^

Brain-derived neurotrophic factor (BDNF) is found throughout the brain and is involved in neurogenesis and neuroplasticity, having a potential role in the pathophysiology of many neuropsychiatric disorders.^5^ Several studies have assessed BDNF levels in various neuropsychiatric disorders (that is, major depression,^6, 7^ bipolar disorder^8^ and schizophrenia^9, 10^), and evidence suggests that BDNF levels are significantly decreased in these disorders. A meta-analysis found no differences in the decrease of peripheral BDNF levels among patients with mood disorders (major depression, bipolar depression and manic episode) and schizophrenia in acute states.^11^ In addition, decreased BDNF levels were observed in mood disorders during acute episodes when compared with those levels in euthymia/remission.^11^ Because most studies have shown a decrease in BDNF^9, 12^ in schizophrenia, BDNF can be considered a schizophrenia biomarker, and may also have a role in the process of neuroprogression in other neuropsychiatric disorders.^13^

However, meta-analyses may be hampered by the heterogeneity of BDNF assays, lack of BDNF standard values and heterogeneity among the populations included in the studies. To address these issues, our study aimed to test, in a ‘true-to-life' setting, the hypothesis that the BDNF serum level nonspecifically decreases in acute SMI patients and increases during inpatient treatment. Until now, there have been few data available about BDNF levels in single samples of patients with different diagnoses, or in patients with SMI in acute clinical presentations.

The objectives of the present study were as follows:

- To evaluate SMI patients' BDNF levels at admission and upon discharge following inpatient treatment at a general hospital.
- To compare BDNF levels among different SMI patient diagnostic subgroups (major depression, bipolar depression, manic episode and schizophrenia).
- To compare BDNF levels in SMI patients at both admission and discharge with those of healthy controls.

## Materials and Methods

Patients were recruited in a general hospital, tertiary inpatient psychiatric unit (Hospital de Clínicas de Porto Alegre, Brazil), and provided written informed consent. Patients aged 18 years or older admitted between June 2011 and December 2013 were invited to participate. Exclusion criteria included insufficient communication skills to participate in the interview or provide written informed consent, and those patients with a primary diagnosis of drug or alcohol dependence. The study was conducted in compliance with the Declaration of Helsinki and was approved by the local ethics committee (GPPG-HCPA id: 100265).

Controls were recruited from a local blood donation center. The healthy control group comprised 100 volunteers who were screened for psychiatric disorders with the Self-Reporting Questionnaire - Brazilian version (SRQ-20).^14^ Those included in the study were not using any psychiatric medication nor had any other general medical condition.

Upon admission to the inpatient unit, patients were screened for eligibility. Patients were included if they had the two SMI criteria of having any mental disorder, with Global Assessment of Functioning (GAF) ⩽50 (in the initial evaluation) and duration of previous services contact ⩾2 years.^3^ Within 72 h of hospitalization, the following clinical evaluations were undertaken: Mini-International Neuropsychiatric Interview (MINI);^15^ Clinical Global Impression scale—Severity (CGI-S),^16^ a clinician-rated seven-point scale that measures disease severity; Brief Psychiatric Rating Scale,^17^ to measure psychiatric symptoms such as depression, anxiety, hallucinations and unusual behavior; GAF,^18^ to measure symptomatology and functioning; Cumulative Illness Rating Scale (CIRS) to evaluate comorbidities.^19^ Clinical and sociodemographic assessments were performed by trained psychiatrists and psychiatry residents. Blood samples were also obtained. Patients were also evaluated 72 h before discharge from the unit, and blood samples were also collected at this time.

### BDNF assessments

Ten-milliliter samples of venous blood were collected into an anticoagulant-free vacuum tube between 1300 hours and 1700 hours. Blood was then centrifuged at 4000 *g* for 10 min and serum was collected and stored at −80 °C. Serum BDNF levels were measured by sandwich enzyme-linked immunosorbent assay (ELISA) in accordance with the manufacturer's instructions (ChemiKine Brain Derived Neurotrophic Factor Sandwich ELISA Kit, Catalogue No. CYT306, Millipore, Billerica, MA, USA). Microtiter plates (96-well flat-bottom) were coated for 24 h at 4 °C with the samples diluted 1:100 in sample diluent. The standard curve ranged from 7.8 to 500 pg ml^−1^ of BDNF. Plates were then washed four times with wash buffer and a biotinylation mouse anti-human BDNF monoclonal antibody (diluted 1:1000 in sample diluent) was added, and incubated for 3 h. After washing, a second incubation was performed with streptavidin-horseradish peroxidase conjugate solution (diluted 1:1000) for 1 h. These procedures were performed at room temperature. After addition of substrate and stop solution, the amount of BDNF was determined (absorbance set at 450 nm). The standard curve showed a direct relationship between optical density and BDNF concentration. The assay range of detection is 15–1000 pg ml^−1^. The manufacturer reports no significant crossreactivity with NGF, NT4/5 or NT3. In addition, intra-assay and inter-assay coefficients of variation are +3.7% (250 pg ml^−1^) and +8.5% (250 pg ml^−1^), respectively. Tests were not performed in duplicate and the investigator was not blinded to the group allocation during the experiment.

### Statistical analysis

All analyses were performed using the Statistical Package for the Social Sciences, version 18 software for Windows (SPSS, Chicago, IL, USA). The normality of the data distribution was examined by the Shapiro–Wilk test. The means were compared between groups using the Student's *t*-test for continuous variables, the *X*^2^-test for categorical variables or one-way analysis of variance when appropriate, followed by the Tukey *post hoc* test when statistical significance was reached.

Generalized estimating equations (GEEs) were used for longitudinal data analysis. GEE can be used in models with non-normally distributed errors and non-balanced data (that is, when there are missing data).^20^ In this study, two GEEs were performed. The first analysis evaluated BDNF levels at admission and upon discharge in SMI patients controlled by diagnostic subgroup (major depression, bipolar depression, manic episode and schizophrenia), age, sex, length of stay and weight. The second analysis compared BDNF serum levels between SMI patients and controls at both time points. As there was only one time point assessment in the control group, BDNF values were repeated in the analysis. Age, sex, weight and the presence of a severe systemic condition were also controlled. The parameters chosen for GEE were as follows: identity link, robust estimator (covariance matrix) and unstructured working correlation matrix. *P*-values less than 0.05 were considered to indicate statistical significance.

The sample size was based on a previously published observational study,^21^ whose subjects were recruited from the same site as ours. To detect a mean difference of 0.5, with an alpha level of 0.05 (two-tailed) and 80% power, 17 in each group (patients and controls) is the required sample size.

Hedges' *g* effect size (ES) was calculated based on differences between the means.^22^ These controlled ESs were interpreted with Cohen's convention of small (0.2), medium (0.5) and large (0.8) effects.^23^

## Results

### Sample characteristics

Sociodemographic and clinical characteristics and treatment profiles of the total SMI patient sample, its diagnostic subgroups, and controls are shown in Table 1. There were no significant differences between diagnostic patient subgroups in years of study, race, proportion of individuals with severe systemic disorders, smoking status, global assessment of functioning at admission or treatment with electroconvulsotherapy. However, there were significant differences between subgroups in the mean age (F:4.33; *P*=0.005), sex proportion (*X*^2^: 25.40; *P*<0.001), body mass index (F=3.64; *P*=0.014), number of previous psychiatric hospitalizations (F: 6.36; *P*<0.001), age of first episode (F:21.95; *P*<0.001), hospitalization length (length of stay) (F: 8.44; *P*=0.005) and CGI-S and Brief Psychiatric Rating Scale scores (F:5.30; *P*=0.012 and F:5.38; *P*=0.001, respectively; Tables 1 and 2). Depressed patients were older at their first episode, and had shorter length of stay and significantly less previous psychiatric hospitalizations than other subgroups. Schizophrenic patients had a greater proportion of male individuals, a greater number of previous admissions and higher CGI-S scores. Pharmacological treatment profiles also differed between groups and are shown in Table 1. The total SMI patient sample and all of the diagnostic subgroups showed significant clinical and functional improvement during the inpatient treatment (Table 2). CGI-S mean difference scores between admission and discharge were 1.8 (*t*-test: 16.4; *P*<0.001) and showed a large ES (ES: 1.72; 95% confidence interval (CI): 1.5, 1.95). For 74 SMI patients (31.3%), BDNF data at discharge were missing.

When comparing the healthy controls and the SMI patients' sample set (Table 1), the control group was found to be younger (F: 17.07; *P*<0.001), and had a smaller proportion of female individuals (*X*^2^=8.4; *P*=0.004).

### BDNF levels

**Figure 1:**
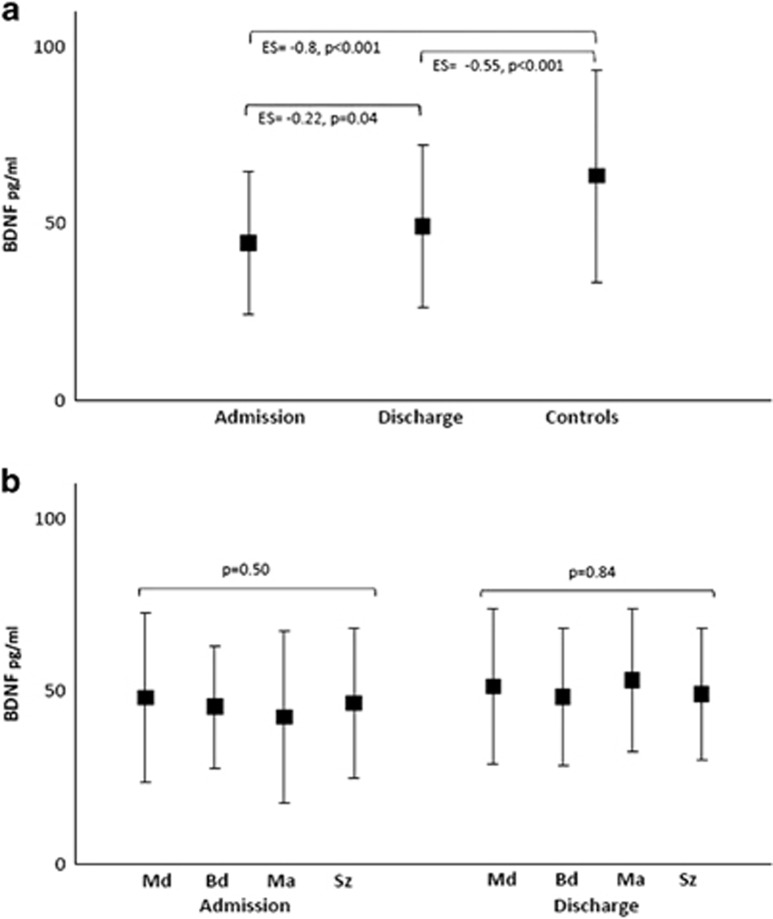
The mean BDNF serum levels in (**a**) SMI patient total sample in admission and upon discharge and in healthy controls and (**b**) in SMI patients subdivided by diagnosis in admission and upon discharge. (**a**) In admission time point, SMI patients' BDNF serum level has a large difference when compared with controls (ES: −0.80; 95% CI: −1.05, −0.56). It increases during the inpatient treatment and remains moderately reduced at discharge when compared with controls (ES: −0.55; 95% CI: −0.81, −0.29). (**b**) BDNF levels among diagnoses are similar in both moments of the treatment. BDNF, brain-derived neurotrophic factor; Bd, bipolar depression; CI, confidence interval; ES, effect size; Ma, mania; Md, major depression; SMI, severe mental illness; Sz, schizophrenia.

The mean BDNF levels increased significantly during the inpatient treatment when SMI patients were analyzed altogether (Figure 1a). However, the ES of the difference between admission and discharge was small (ES: −0.22; 95% CI: −0.43, 0.00). BDNF levels showed significant reductions compared with controls both at admission and upon discharge (Figure 1a). These reductions indicate large to moderate ESs (ES: −0.80; 95% CI: −1.05, −0.56 and ES: −0.55; 95% CI: −0.81, −0.29, respectively). Age, sex, weight and the presence of a severe general medical condition (in SMI patients) did not significantly correlate with BDNF levels.

In addition, BDNF levels were not significantly different between SMI patients from different diagnostic subgroups at both time points of the treatment (Figure 1b). Estimated serum BDNF means (s.d.) at admission and upon discharge were 48.1(24.5) and 51.3(22.6) in major depression; 45.4 (17.7) and 48.4 (20.0) in bipolar depression; 42.5 (20.7) and 53.1 (25.1) in manic episode; and 46.5 (21.8) and 49.1 (19.0) in schizophrenia (Table 3). Age, sex, weight and length of stay were not statistically significantly correlated with BDNF levels in the GEE model.

Pharmacological interventions were not included in the analysis because the sample was composed predominantly of patients with combinations of multiple treatments. Furthermore, the use of mood stabilizers and antipsychotics showed correlation, indicating collinearity. Treatment with electroconvulsotherapy during the hospitalization was evaluated in the first GEE analysis. However, electroconvulsotherapy did not significantly correlate with BDNF levels and was not included in the model.

## Discussion

The three main findings of this study are as follows: (1) SMI patient serum BDNF levels significantly increase during inpatient treatment and increased levels are associated with clinical improvement; (2) estimated mean BDNF levels show no difference among SMI patients with major depression, bipolar depression, manic episode and schizophrenia; and (3) BDNF levels persist at a lower level compared with controls even with the general improvement of clinical scores during the inpatient treatment.

In this naturalistic study, we believe we are the first to replicate in single samples obtained from 336 individuals (236 SMI patients in an inpatient setting and 100 controls) the findings of one previous meta-analysis,^11^ which showed that peripheral BDNF levels were equally reduced in acute presentations among different diagnoses. Furthermore, our results indicate a similar reduction in BDNF levels in acute presentations.

Other meta-analyses of studies of individual disorders have shown significantly reduced peripheral BDNF levels in major psychiatric disorders. A meta-analysis of BDNF levels in major depression showed that BDNF levels were substantially lower when comparing healthy controls with antidepressant-treated depressed patients.^6^ Similarly, serum BDNF levels were significantly lower than in healthy controls in other studies that assessed BDNF levels in depressive episodes.^6^ Furthermore, another meta-analysis that studied manic episodes showed that BDNF levels in manic patients were significantly lower than in controls.^8^ In addition, in schizophrenia, a meta-analysis showed that peripheral serum and plasma BDNF levels are moderately reduced when compared with controls.^9^

Nevertheless, the increase in BDNF levels during treatment is not so pronounced in our total sample. The lack of change in BDNF subgroups was probably due to BDNF unspecificity among different diagnoses and sample size effects. BDNF levels increase but the non-restoration may reflect the characteristic disorder progression observed in SMI patients.^24^ These findings support the suggested role of BDNF in neuroprogression, a phenomenon whereby the central nervous system pathologically reorganizes during the course of a SMI.^25, 26^ The SMI concept brings about a link with disease-staging models, where the SMI patients show a middle-to-late stage of an underlying disease process. In these stages, patients usually have a poor outcome, with heavy medical comorbidity, treatment-refractory symptoms and severely impaired functioning. ^24^ Thus, SMI patients represent an end stage or final phenotype of the psychiatric disorders.

Further studies are necessary to determine BDNF levels over time. The SMI definition is based on the disorder duration, that is, contact with services for 2 years or more. Interestingly, a 2-year longitudinal study showed a more profound decrease in serum BDNF levels in patients with persistent and remitted depression than in non-depressed controls.^27^

The limitations of our findings should be addressed. First, there were differences in some variables between controls and SMI patients. However, these variables showed no significant contribution to the analysis. Second, some data were missing, and this is why GEEs were chosen. Third, BDNF was measured within a short interval of time and we could not assure the stability of the changes. Fourth, correlations between baseline and follow-up BDNF levels were low, indicating a high between-subjects variability of change in serum BDNF over time. Unfortunately, the poor reproducibility of BDNF measures has to date prevented its validation for clinical purposes.^28^ Last, pharmacological treatments were not included in the analysis as most patients used multiple combinations of different pharmacological interventions. Furthermore, recent evidence shows that medication use does not affect BDNF serum levels as much as initially thought.^9^

## Conclusion

The similar reduction observed in BDNF levels among SMI patients with different diagnoses and the significant increase but non-restoration during inpatient treatment indicate that BDNF serum levels could be considered a marker for the presence of an unspecific psychiatric disorder and possibly a transdiagnostic and unspecific marker of disease activity.

## Figures and Tables

**Table 1:** Total sample sociodemographic, clinical and treatment characteristics (controls, severe mental illness patients and subgroups divided by diagnosis)

*Variables*	*SMI* n=*236*	*Controls* n=*100*	*F/X*^*(*2*)*^	P	*SMI*				*F/X*^*(*2*)*^	P	Post hoc
					*Md* n=*110*	*BD* n=*21*	*Ma* n=*43*	*Sz* n=*62*			
Sex, F	134 (56.7)	44	8.4	0.004	74 (67.2)	14 (66.6)	27 (62.7)	19 (30.6)	25.40	<0.001	(Ma=Bd=Md)>Sz
Age, years	44.8 (14.1)	33.6 (10.9)	17.0	<0.001	47.8 (13.6)	49.3 (11.8)	42.0 (17.1)	40.9 (12.0)	4.33	0.005	(Md>Sz)=Ma=Bd
Years of study	9.7 (4.5)	12.3 (3.8)	13.9	<0.001	10.0 (4.6)	10.3 (4.5)	10.7 (4.4)	8.9 (4.5)	1.96	0.143	—
Race, white	204 (86.4)	83	0.66	0.414	68 (80.0)	20 (95.2)	36 (80.0)	58 (87.9)	—	0.286	—
BMI	27.1 (5.6)	26.3 (3.3)	3.1	0.116	26.7 (5.3)	31.0 (6.3)	26.8 (6.1)	26.6 (5.3)	3.64	0.014	Bd>(Md=Sz=Ma)
Systemic disorder, yes	17 (7.2)	—	—	—	11 (12.4)	0	2 (4.7)	4 (6.5)	5.03	0.169	—
Smokers, yes	62 (26.2)	18	2.2	0.104	25 (34.2)	8 (44.4)	14 (36.8)	15 (27.7)	1.94	0.583	—
Previous admissions, *n*	4.3 (4.2)	—	—	—	2.64 (3.7)	3.2 (3.8)	4.3 (4.2)	7.3 (10.9)	6.36	<0.001	(Sz=Ma=Bd)>Md
Age of onset	29.4 (13.3)	—	—	—	34.2 (13.6)	33.3 (10.8)	26.4 (14.7)	23.3 (9.4)	21.95	<0.001	(M=Sz)<(Md=Bd)
LOS, days	30.8 (21.4)	—	—	—	25.3 (11.6)	30.9 (15.4)	32.0 (21.5)	37.7 (30.3)	8.44	0.005	(Ma=Bd=Sz)>Md
											
*Treatment options during inpatient stay*											
Antidepressants, yes											
Admission	78 (33.5)	—	—	—	53 (48.2)	6 (28.6)	9 (20.9)	10 (16.1)	22.45	<0.001	Md=Bd>(Sz=Ma)
Discharge	89 (37.7)	—	—	—	77 (71.3)	11 (52.4)	6 (14.0)	6 (9.7)	79.04	<0.001	(Md=BD)>(Sz=Ma)
Mood stabilizers, yes											
Admission	80 (33.8)	—	—	—	36 (32.7)	9 (42.9)	18 (41.9)	17 (27.4)	3.19	0.362	—
Discharge	79 (33.9)	—	—	—	32 (31.4)	14 (66.7)	33 (76.7)	16 (25.8)	38.03	<0.001	(Ma=Bd)>(Md=Sz)
Antipsychotics, yes											
Admission	141 (59.7)	—	—	—	59 (53.6)	13 (61.9)	26 (60.5)	43 (69.4)	4.13	0.247	—
Discharge	199 (84.3)	—	—	—	82 (74.5)	17 (81.0)	39 (90.7)	61 (98.4)	17.09	0.001	(Sz=Ma)>(Md=Bd)
ECT	68 (28.8)	—	—	—	28 (31.8)	10 (47.6)	8 (18.6)	18 (29.0)	5.93	0.115	—

Abbreviations: BD, bipolar depression; BMI, body mass index; ECT, electroconvulsotherapy; F/X2, F statistics value/X2-statistics value; LOS, length of stay; Ma, mania; Md, major depression; SMI, severe mental illness; Sz, schizophrenia.

Values are shown as mean (s.d.) or *n* (%).

**Table 2:** Functional and clinical variables in admission and upon discharge

*Variables*	*SMI* n=*236*	*SMI*				F	P	Post hoc
		*Md* n=*110*	*BD* n=*21*	*Ma* n=*43*	*Sz* n=*62*			
*GAF*								
Admission	34.1 (14.6)	34.5 (15.2)	40.0 (13.3)	34.7 (15.0)	31.2 (13.6)	1.98	0.118	—
Discharge	60.2 (16.7)	66.4 (12.8)	56.0 (19.2)	60.2 (18.1)	52.9 (16.7)	5.15	0.002	Sz<(D=Bd=Ma)
								
*BPRS*								
Admission	23.9 (10.8)	21.3 (9.8)	20.3 (7.4)	26.5 (11.2)	27.5 (11.7)	5.38	0.001	(Md=Bd=Ma)<Sz
Discharge	9.6 (7.3)	7.8 (5.6)	11.1 (8.3)	7.7 (6.1)	12.9 (8.4)	5.48	0.001	(Md=Bd=Ma)<Sz
								
*CGI-S* *score*								
Admission	5.3 (0.97)	5.1 (1.00)	5.1 (0.8)	5.5 (0.87)	5.5 (0.95)	5.30	0.012	(Ma=Bd=Md)<Sz
Discharge	3.4 (1.23)	3.0 (0.94)	3.4 (1.14)	3.3 (1.52)	4.1 (1.15)	6.55	<0.001	(Md=Bd=Ma)<Sz

Abbreviations: BD, bipolar depression; BPRS, Brief Psychiatric Rating Scale; CGI-S, Clinical Global Impression Severity Scale; F, F statistics value; GAF, Global Assessment of Functioning; Ma, mania; Md, major depression; SMI, severe mental illness; Sz, schizophrenia.

**Table 3:** Serum BDNF in admission and upon discharge among diagnostic subgroups

*SMI subgroup*	*BDNF (pg ml^−1^)*		*Wald* X^*2*^	P	*ES*	P
	*Admission*	*Discharge*				
Major depression	48.1 (24.5)	51.3 (22.6)	0.887	0.357	0.13	0.418
Bipolar depression	45.4 (17.7)	48.4 (20.0)	0.382	0.536	0.16	0.592
Mania	42.5 (20.7)	53.1 (25.1)	3.464	0.063	0.44	0.084
Schizophrenia	46.5 (21.8)	49.1 (19.0)	0.641	0.423	0.12	0.544

Abbreviations: BDNF, brain-derived neurotrophic factor; ES, effect size; SMI, severe mental illness.
